# Internet of Things (IoT): Sensors Application in Dairy Cattle Farming

**DOI:** 10.3390/ani14213071

**Published:** 2024-10-24

**Authors:** Francesco Maria Tangorra, Eleonora Buoio, Aldo Calcante, Alessandro Bassi, Annamaria Costa

**Affiliations:** 1 Department of Veterinary Medicine and Animal Science, University of Milan, Via dell’Università, 6, 26900 Lodi, Italy; francesco.tangorra@unimi.it (F.M.T.); annamaria.costa@unimi.it (A.C.); 2 Department of Agricultural and Environmental Sciences—Production, Landscape, Agroenergy, University of Milan, 20133 Milan, Italy; aldo.calcante@unimi.it; 3 Eurescom GmbH, Wieblinger Weg 19/4, 69123 Heidelberg, Germany; abassi@iotitaly.net

**Keywords:** precision dairy farming, smart farming, farm automation, data-driven decision

## Abstract

The expansion of dairy cattle farms and the increase in herd size have made the control and management of animals more complex, with potentially negative effects on animal welfare, health, productive/reproductive performance and consequently farm income. Precision Livestock Farming (PLF) is based on the use of sensors to monitor individual animals in real time, enabling farmers to manage their herds more efficiently and optimise their performance. The integration of sensors and devices used in PLF with the Internet of Things (IoT) technologies (edge computing, cloud computing, and machine learning) creates a network of connected objects that improve the management of individual animals through data-driven decision-making processes. This paper illustrates the main PLF technologies used in the dairy cattle sector, highlighting how the integration of sensors and devices with IoT addresses the challenges of modern dairy cattle farming, leading to improved farm management.

## Simple Summary

The use of automation and sensor-based technologies has the potential to enable dairy farmers to control and manage their herds better and in less time, based on a wealth of information about the production process, the herd and the individual animal. However, the effectiveness of this information depends on its effective integration into the decision-making process. Today, the proliferation of ever-larger dairy cattle farms makes it difficult to monitor individual animals directly in the herd, with the risk of compromising their health, welfare, and production performance, which will inevitably affect the farm budget. The Internet of Things (IoT), a system of devices and sensors that communicate via cloud computing, could narrow this gap and open up new opportunities for dairy farmers.

## 1. Internet of Things

### 1.1. Concept and Vision

Although the expression “Internet of Things” (IoT) is widely used today, there has always been some fuzziness about its precise definition. The challenge lies in the fact that IoT represents a convergence of various concepts and technologies—such as sensor networks, cloud data, and smart devices—without clearly defined boundaries. Initially, the idea of IoT was centred on the connectivity of devices, focusing on linking objects to the internet, allowing basic data collection and remote control. For example, sensors would collect data from objects like thermostats or refrigerators, sending information to a centralised platform for monitoring or remote operation. However, more recently, the expression has expanded into a far more complex and integrated ecosystem, with a key shift towards intelligence and autonomy. Nowadays, IoT systems often incorporate advanced analytics in order to transform simple data into intelligence, allowing automated decision-making without human intervention.

Additionally, the scope and scale of IoT networks have grown dramatically. Early IoT implementations involved a small number of connected devices in limited contexts, such as home automation or logistics tracking. Today, IoT is applied across a variety of sectors, from healthcare and agriculture to smart cities and industrial systems.

The shift towards intelligence also meant a much higher focus on edge computing. Initially, IoT data would be sent to centralised cloud servers for processing. With the increasing number of devices and the need for faster real-time decision-making, the need for systems able to process data closer to where the data is generated has greatly increased to reduce latency and improve performance, particularly for time-sensitive applications.

Therefore, twenty years ago, IoT could have been correctly described by the International Telecommunication Union [1] as a connection between product identification and sensing technologies and their capacity to engage with the surroundings. From that perspective, the “Internet” is described as “the worldwide network of interconnected computer networks, based on a standard communication protocol, the Internet suite (TCP/IP)”, while a “Thing” is defined as “an object not precisely identifiable”, making IoT, “a worldwide network of interconnected objects uniquely addressable, based on a standard communication protocol” [2]. This definition, today, is missing the more contemporary aspects; therefore, we can summarise the current IoT concept with three key characteristics [3]:

1. It creates a digital representation of a physical entity, often referred to as a digital twin.
2. It involves the use of various sensors and can alter the environment through actuators.
3. It has the capability to perform at least some level of information processing.

**Figure 1:**
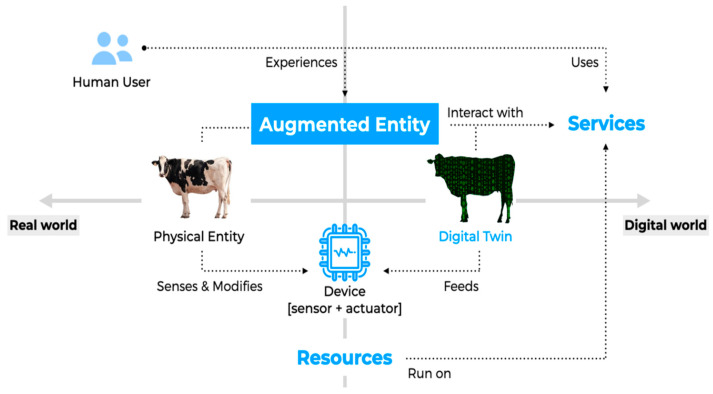
Domain model for the Internet of Things. The solid lines indicate the transposition from real to digital world and the direct actions or processes. Dashed lines represent interactions (e.g., user services, device data and resources). Blue words highlight key components.

In summary, the Internet of Things can be described as a physical entity paired with its digital counterpart interacting through various devices. This combination of physical and digital creates an augmented entity that supports services accessed by end users, typically humans. These services run on resources that are directly connected to the devices (Figure 1).

From a functional perspective, various functional models have been proposed to define how IoT functional blocks operate across their different layers and use cases. These models help to understand the underlying architecture and enable the design of IoT systems that can efficiently meet the demands of diverse applications.

Initially, a three-layer model was proposed consisting of the following:

- Perception Layer: This is the physical layer that includes all the sensors and devices that collect data from the environment. The primary function is to gather information through IoT devices and sensors.
- Network Layer: Responsible for transmitting the data collected from the perception layer to other layers or systems.
- Application Layer: Where the data is processed and utilised by applications, providing services to users.

While this model is simple and clear, it is now considered limited as IoT has grown more complex. To capture the growing complexity of IoT environments, two more layers were introduced:

- Processing Layer: Also known as the middleware layer, this handles data storage, processing, and management. This layer might involve cloud computing and big data platforms that perform analytics or decision-making.
- Business Layer: This layer focuses on managing and orchestrating the IoT system as a whole, including policies, privacy, and business models. It helps ensure that the IoT deployment aligns with organisational goals and provides value.

The ITU-T introduced a more comprehensive model with seven layers. This model is designed to offer a comprehensive look at all aspects of IoT, from connectivity to data usage:

- Device Layer: Where sensors and actuators reside.
- Network Layer: Manages communication between IoT devices and the back-end systems.
- Service Support and Application Support Layer: Provides the necessary computing power and storage for IoT services.
- Service Layer: Enables different services and applications to run efficiently over the infrastructure.
- Application Layer: Focuses on delivering services to end users.
- Management Layer: Responsible for managing all resources, ensuring scalability, and monitoring the network’s health.
- Security Layer: Ensures data protection, privacy, and secure communication across the system. This layer has become more prominent with the increasing focus on cybersecurity in IoT deployments.

**Figure 2:**
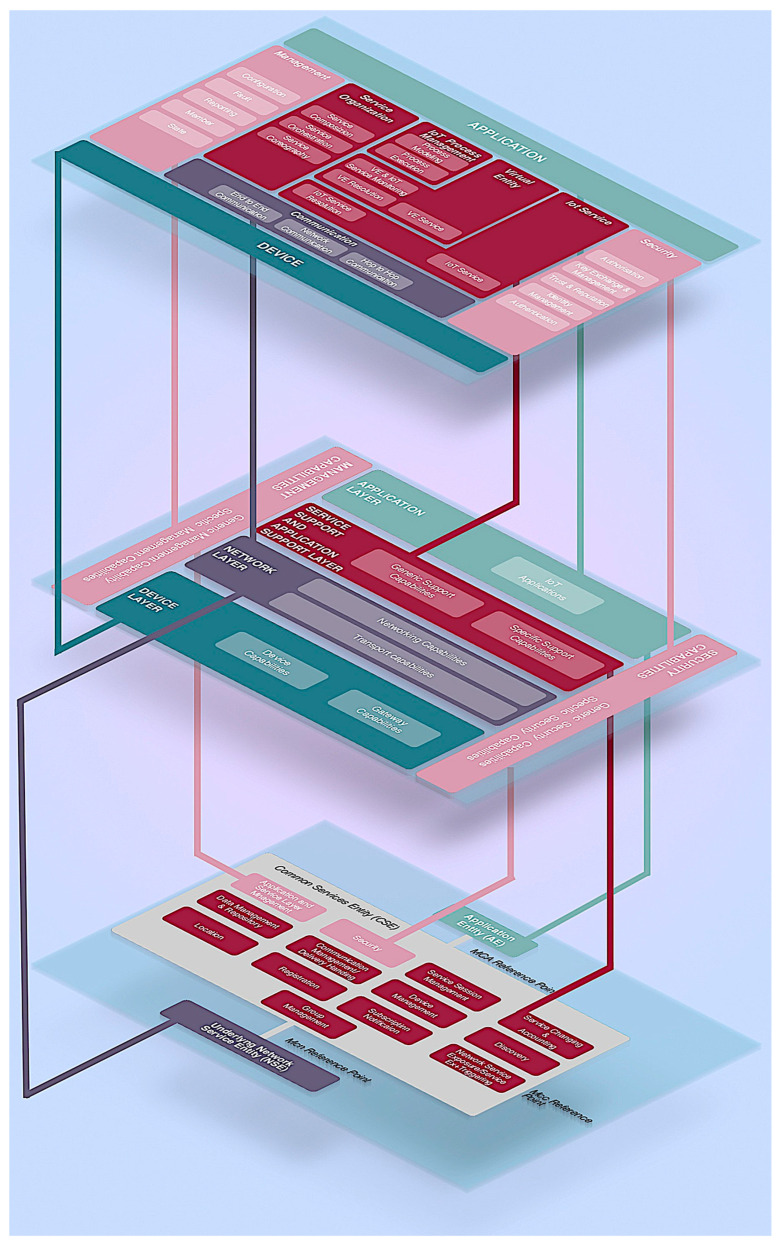
The similarities between different IoT functional models (IoT-A, ITU-T and oneM2M).

It is important to note that multiple initiatives are currently working in parallel to develop a comprehensive reference model for the IoT ecosystem. Among these efforts are IoT-A [4], IEEE P2413 [5], ITU-T [6], IIC [7], and oneM2M [8]. While these frameworks often employ varied terminologies, the underlying concepts are largely consistent across them. For example, as shown in Figure 2, the functional models of IoT-A, ITU-T’s reference architecture, and oneM2M’s functional architecture are quite comparable in their structure and approach.

In the context of smart dairy farming, deploying Internet of Things (IoT) devices equipped with sensors represents a pivotal aspect of monitoring livestock health and productivity. Wearable sensors attached to cows track parameters, allowing for the collection of real-time data on each animal’s health status.

Recent advances like artificial intelligence, computer vision algorithms such as YOLO (You Only Look Once) v9, and automated sensors have been introduced, for example, to characterise the barn environment and assess cow behaviour in relation to it [9] and to detect changes in health indicators [10], highlighting the potential of these technologies for more effective herd monitoring and management. These technologies have the advantages of being non-contact and therefore non-stressful, with low cost and high yield potential [11].

In light of the potential for transformative change offered by the Internet of Things (IoT) in agriculture, particularly in dairy farming, undertaking a comprehensive review of the technologies currently available in this sector is of great importance. This review aims to collate and assess the numerous advancements in smart farming, emphasising integrating IoT solutions and their implications for economic sustainability. By analysing the current landscape of these technologies, insights can be provided into how they enhance operational efficiency, improve herd management and support animal welfare.

A deep understanding of the developments under this framework will inform farmers about the management of resources and cost reduction. Besides that, this review reports a reflection on the economic sustainability of intelligent farming technology as an avenue for enhancing production with the certainty of the long-term viability of dairy farms. As a matter of fact, farmers in the competitive market are compelled to adopt such improvements to fulfil their economic and environmental responsibilities and enhance performance.

### 1.2. IoT and Dairy Cattle Farming

IoT and data-driven techniques can provide new opportunities for dairy farmers to manage their farms. IoT acts as a bridge between virtual and physical domains, focusing on wireless communication through smart devices such as sensors that use local and global infrastructures to connect and enable fully autonomous operation of IoT systems [12,13]. This creates an on-farm network consisting of sensors on or in the body of the dairy animal connected to other points in the farm [14], which requires an efficient communication system using multiple protocols. The integration of sensors with network technology has led to the development of sensor nodes [15], entities that can generate data (edge), process or transform data (fog) and store data (cloud). An example of how this network works is the real-time monitoring of body temperature changes in dairy cows. In this case, the temperature sensor in the rumen, which is both the data source and the farthest node in the network (edge node) from the central hub, sends data to the collar, which has computational and analytical capabilities, such as pre-processing data and identifying behavioural patterns, making it a fog node in the network. Finally, the data are uploaded through the network gateway to remotely accessible cloud storage, where subsequent actions such as analysing body temperature data through deep learning algorithms to detect disease, oestrus, parturition, or mastitis become possible [16,17]. This architecture is particularly suited to dairy farms using large-scale wireless sensor networks, where large amounts of data are generated and need to be transmitted. The computational and analytical capabilities of edge and fog nodes allow low-level devices to process and act on data as they are generated, providing a decentralisation of data processing and decision-making that results in low-latency and efficient networks [18]. Recently, fog and edge computing infrastructures have been incorporated into several systems developed for animal health monitoring and management [19,20,21,22,23].

Reduced data redundancy, reduced data transmission, more self-sufficient and robust networks, and lower bandwidth requirements are the main advantages of fog and edge computing-based livestock monitoring and management systems. Limitations to the adoption of edge and fog computing on livestock farms include the cost of specialised edge devices available on the market. They also require regular updates or replacements over time, increasing the investment for the dairy farmer. On the other hand, the use of open-source solutions such as Raspberry Pi (Raspberry Pi Foundation, San Francisco, CA, USA) and Arduino (https://www.arduino.cc/, accessed on 14 October 2024) requires expertise that is not easy to find on dairy farms [17].

The adoption of IoT-based sensors on dairy farms is rather heterogeneous, with some sensors, such as those for measuring cow activity and milk production, being widely used and others, such as those for lameness detection and body condition score assessment, being newly adopted [24,25,26,27,28]. A critical factor in adoption is user acceptance, which involves a complex and dynamic decision-making process influenced by contextual factors, perceptions and social conditions. Acceptance depends on both cognitive (e.g., cost-benefit analysis) and affective (e.g., emotions and trust) factors, the role of which in shaping adoption decisions is not yet fully understood. The complexity of the adoption process suggests that strategies to improve adoption should address both rational and emotional factors, as well as technical and infrastructural barriers, to promote greater integration of IoT-based sensors in dairy farming.

High investment, initial installation and maintenance costs are perceived as the main barriers to the adoption of IoT technologies on dairy farms, while compatibility issues with existing systems hinder wider adoption. Infrastructure challenges, such as limited access to broadband, further complicate the adoption process. Farmers’ confidence in the reliability and perceived benefits of these technologies also affect adoption [29,30].

IoT systems enable large-scale data collection and monitoring, incorporating a wider range of parameters, while cloud computing and machine learning facilitate high-precision simulations. These innovations are expected to drastically reduce both the time and cost associated with developing models and decision support systems necessary to improve livestock farm management [31]. However, in the agricultural sector, the implementation of IoT infrastructure faces substantial challenges, primarily due to the variability of production cycles and the remote nature of many farms. In fact, the high cost of deploying such infrastructure, particularly in isolated areas, is often prohibitive, especially for smaller-scale farmers with limited financial resources.

IoT can automate labour-intensive tasks, such as real-time health monitoring of livestock, thereby reducing labour costs by delivering individualised, real-time data. In livestock management, where feed and disease control are the primary costs, advanced technologies such as AI and machine learning algorithms can optimise decision-making processes related to feed rates, diet formulation, and treatment plans [32]. Repeated daily operations (milking, cleaning, etc.) may also have a reduced economic impact due to lower labour requirements [33].

Despite these benefits, although there are low-cost sensors available on the market, the high upfront investment required for IoT adoption remains a critical barrier [13,34]. The financial burden associated with implementing IoT in Precision Livestock Farming (PLF) is compounded by ongoing maintenance and data management expenses. Although the potential advantages—such as labour savings, improved resource efficiency, and enhanced environmental sustainability—are widely acknowledged, the uncertainty surrounding return on investment (ROI) complicates the decision-making process for farmers. This is especially significant in agricultural operations with narrow profit margins, where the costs of IoT devices and integrated systems may outweigh the potential financial benefits [35].

Additionally, poor internet connection in rural areas and long-term data management further exacerbate the costs and complexity of IoT implementation. Although technological advancements are expected to reduce costs and improve accessibility in the near future, the economic viability of IoT adoption in agriculture remains a major concern. Rigorous cost-benefit analyses and case studies are necessary to provide farmers with the insights required to make informed decisions regarding IoT investments [36].

In summary, while IoT technologies hold substantial promise for transforming agriculture and livestock management, the high costs of implementation—including infrastructure deployment, sensor maintenance, and data management—represent significant barriers to their widespread adoption [13]. Addressing these financial challenges through technological innovation, policy initiatives, and comprehensive research is essential to ensuring the sustainable integration of IoT into agricultural and zootechnical practices.

## 2. Dairy Farm Automation

Automation of the production processes in dairy farming is rising throughout the world. In general terms, automation refers to using machines, control systems, and information technologies to enhance productivity in the production processes [37]. The major drivers of this change are the reduction of physical labour and labour costs [38,39].

The application of automation fits with the trend of fewer but larger herds, narrower profit margins than in the past, and continuous improvement of technology already available that becomes less costly [40,41,42]. Automation allows collection of a large amount of data from the monitored animals and the surrounding environment. After an appropriate elaboration process, data provide helpful information for farmers to control and manage herds by allowing them to make the right decisions [25].

This latter aspect plays a key role in dairy farm management, encouraging a proactive rather than a reactive approach. However, this is dependent on each farmer’s individual skills. While automation and technology may not solve problems directly, they can identify areas that need attention. By adopting this perspective, automation can make a positive contribution to profitability, animal welfare, milk quality, and overall lifestyle improvements.

**Figure 3:**
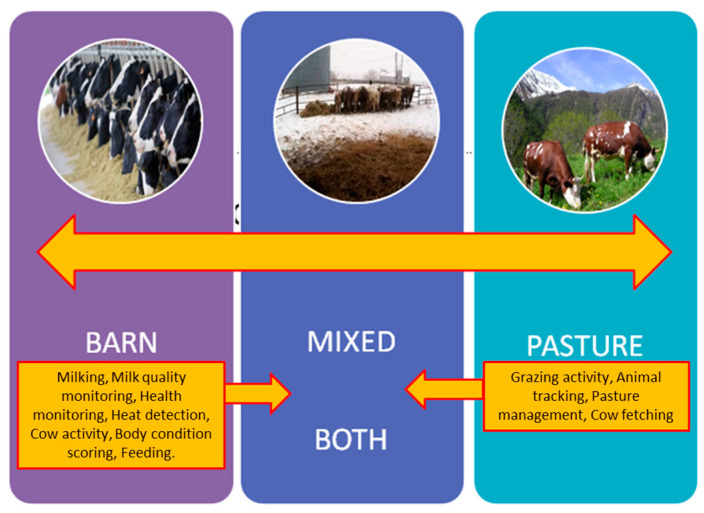
Main processes and tasks that can be potentially automated and monitored in dairy cattle, depending on the rearing system.

An important aspect to consider in automating the dairy cattle sector is the different rearing methods available: enclosed facilities, where cows are essentially kept in barns; grazing; and the mixed system, which is a combination of the aforementioned two. The utilisation of these technologies is more prevalent in closed farms, whereas their adoption in pasture systems is comparatively limited. An illustrative example of pervasive technology in grazing-based dairy farms includes heat detection sensors and chew sensors, which record mandibular activity, allowing the assessment of forage availability in the area where animals are kept [43]. Figure 3 summarises the main processes that can be automated and monitored using specific sensors.

Considering the barn solution, dairy farm automation usually concerns two main areas: (i) Animal IoT sensors and (ii) Farm IoT sensors.

### 2.1. Animal IoT Sensors

Continuous on-farm monitoring of livestock using IoT sensors enables early detection of health problems, optimisation of feeding regimes and improvement of animal welfare, reducing veterinary costs, increasing productivity and improving the quality of dairy products.

The sensors used in these systems can be attached or detached from the cow, depending on the specific requirements of the monitoring process. The attached sensors can be external, as in the case of on-cow sensors (e.g., pedometers) or internal, as in the case of in-cow sensors (e.g., rumen boluses). Sensors detached from the cow are called off-cow sensors and can be divided into two categories: (i) in-line sensors work by continuously monitoring variables in the milk stream (e.g., milk electrical conductivity); (ii) online sensors are equipped to automatically collect and analyse milk samples, such as those used to determine somatic cell count [42].

In addition to these sensors, instruments based on image and sound have emerged as a novel approach in the field of dairy farming. Cameras that are equipped with data processing tools are able to scan moving objects and analyse a number of features, including posture, walking speed and gait. This allows for the diagnosis of various health issues in animals, such as sore legs, sickness and emaciation. Similarly, sound-based sensors can analyse vocalisations and environmental sounds to detect indications of distress, respiratory issues, or alterations in behaviour.

The integration of these sophisticated technologies serves to enhance the precision of health monitoring, offering a more real-time comprehension of the animal’s status.

#### 2.1.1. Sensors for Body Measurement

Body measurements, such as Body Condition Score (BCS) and Body Weight (BW), are crucial for making informed management decisions, such as refining breeding strategies, evaluating nutritional health, and tracking daily weight gain [44].

BCS is an indicator of weight loss and gain in the early lactation and pre-calving periods, respectively. In the first case, it is useful for limiting metabolic disorders, while in the second, it is useful for managing dry cows. It is based on a visual method carried out by experts and provides an assessment of the animal’s energy reserves at different stages of lactation [44,45]. The Edmond scale [27] assigns a score between 1 (underweight animal) and 5 (overweight animal) to a cow based on the assessment of specific anatomical regions of the body. A score of 3–3.5 corresponds to a healthy condition.

In order to automate the analysis of the BCS index, a series of experimental trials were conducted, wherein the BCS value was typically defined through a model based on image analysis [28]. In particular, Peacock et al. [46] and Bewley et al. [29] used a 2D camera. A thermal camera was applied by Halachmi et al. [30] to extract the cow’s body shape from its background and to evaluate, with an opportune algorithm, the BCS of a single cow. Spolianski et al. [47] and Calcante et al. [48] proposed a system based on low-cost 3D cameras to estimate the BCS automatically. Kuzuhara et al. [49] and Gomes et al. [50] also used the same technique to develop models for BW assessment.

Body weight can be defined by analysing the lateral or dorsal image of a cow. These models have a commendable evaluation capability. In the first case, BW (kg) is defined based on measurements of height (m), body depth (m) and lateral body volume (m^3^), while in the second it is calculated by trunk (m), thorax (m) and dorsal area (m^2^) [44].

Implementing a fully automated system still presents some challenges, particularly regarding image quality and processing [44].

However, in this way, the farmer can monitor the herd at the most appropriate time with high precision and objectivity and can modify the feeding of a single cow by acting on the feeding system in terms of the quantity and quality of the ration.

#### 2.1.2. Sensors for Activity Monitoring

Monitoring the cow’s basic behaviours (eating, rumination, standing, lying, walking, drinking, and mounting) allows one to ascertain the cow’s overall health status [51]. Monitoring animal behaviour activity patterns allows early detection of specific conditions such as lameness, thermal discomfort, oestrus or calving events, and diseases. For example, some studies have shown that podiatric pathologies are associated with changes in walking speed [52] and altered leg swing patterns [53].

Lameness can also be detected through a pressure-sensor mat, which is capable of detecting alterations in footfall and weight distribution, crucial indicators of potential foot-related issues [54]. The advent of lameness sensors represents a substantial advancement in the sustainable and holistic management of dairy herds. The sensors transmit real-time data to farmers, enabling prompt intervention when necessary.

This method of monitoring behaviour improves performance and animal welfare and makes it easier to make decisions and respond to situations [55].

Several types of sensors exist for this purpose, usually integrated with collars, leg bands, or ear tags. However, the most common animal-related sensors are pedometers and accelerometers for automatic oestrus detection, such as those produced by SCR by Allflex (Netanya, Israele), Nedap (Groenlo, Paesi Bassi) and DeLaval (Tumba, Svezia) [56].

Pedometers are electronic devices attached to the cow’s leg that can record the cow’s movements: with each step taken by the animal, the device increments a counter in the internal memory. The final daily step count and the cow’s identification code are transmitted to a receiver after the animal has been identified by an antenna in the barn [57]. If the animal’s activity exceeds a user-defined threshold, an alarm is generated at a specific time to advise the farmer to proceed with artificial insemination [58].

Accelerometers are attached to the cow’s neck collar and measure accelerations associated with head and neck movements during walking and herding behaviour. A triaxial accelerometer allows the collection of three-dimensional information and gravity [59].

Data are read by an antenna placed near the milking system and transmitted via IR signal to the herd management software. Through the comparison of the currently measured data with the stored activity pattern, the cow’s daily activity is separated from activities associated with oestrous behaviour using specially developed algorithms. The herdsman receives an alert when cows exceed a user-defined threshold [60,61].

Various parameters, including neck activity, ear movement, leg position and activity, the number of steps, the duration and frequency of rest intervals, rumination behaviour, feeding times and reticulum temperature, can be used to indicate the onset of oestrous. The application of machine learning techniques to collect data automatically appears promising in identifying the onset of oestrous [62]. The advantages of automatic detection of oestrous compared to visual observation have been demonstrated, allowing a higher heat detection rate and improving fertility index in dairy cattle herds [63]. Enhanced detection accuracy translates to reduced wastage of insemination, time-saving and diminished economic loss [64].

#### 2.1.3. Sensors and Systems for Calving Monitoring

Calving time in dairy farms can be crucial, especially for primiparous cows, as it can lead to complications and trauma for the cow and calf [65]. Difficult delivery can decrease milk production, cause uterine infection, increase veterinary intervention expenses, and potentially cause infertility in the cow, followed by premature culling [66].

Accurate calving prediction can reduce potential health risks to the calf from the mother or the environment [67]. This is more critical for cows with first-calving problems and those producing high-value calves, such as those obtained by embryo transfer [68]. Moreover, uncertainty in determining the exact time of birth reduces the chances of timely intervention [69].

The use of sensors in animals to detect parturition is well documented in the scientific literature. Most studies use accelerometers in collars, ear tags or pedometers. These devices can measure behavioural changes associated with parturition, such as a decrease in rumination and the duration of the decubitus position [70]. Various methods have also been evaluated to predict calving time by measuring changes in body temperature [71,72], ultrasound [73], blood levels of estrone sulphate and 17-b-estradiol [74] or progesterone [68,75]. Other methods include observing the progress of relaxation of the pelvic structure [76] and measuring electrolyte levels in milk [77]. However, these methods are often time-consuming and expensive. An alternative method is the intravaginal sensor, which is placed near the cervix. At the moment of parturition, the sensor is released by detecting changes in temperature and light. These changes trigger an alarm to the farmer [78]. This sensor can be particularly useful in pasture and intensive farming where parturition takes place in specific, confined areas. Accurate prediction of calving time is of paramount importance for cows reared in extensive grazing areas. The large area makes it difficult to intervene in time to prevent adverse calf outcomes caused by the parturient cow or an adverse environment. To address this, Calcante et al. [79] developed a GPS/GSM calving alarm system, the GPS-CAL (GPS-Calving Alarm). This device accurately predicts calving time and sends a text message to the farmer with the date and time of calving, animal ID and GPS coordinates of the calving location. These coordinates are in a Google Maps compatible format, allowing the farmer to easily locate the calving location using a mobile phone application.

#### 2.1.4. Sensors for Mastitis Detection

Mastitis in dairy cows has a significant impact on animal health and welfare, leading to reduced milk yield and quality. This condition results in significant economic losses due to veterinary interventions, medical treatments and, in severe cases, culling [80].

The electrical conductivity (EC) of milk has been identified as a critical indicator for the early detection of mastitis in dairy cows. To this end, several researchers have proposed predictive models based on time series analysis of EC measurements and comparison with EC values from different quarters during the milking process [81,82,83,84,85,86]. The possibility to measure EC continuously and automatically during milking by means of electrodes integrated into the milking unit makes this parameter particularly useful for early detection of mastitis, especially when combined with milk yield and average milk flow [87]. Although studies have confirmed that the EC of milk from cows with both clinical and subclinical mastitis is significantly higher than that of healthy cows [83,86], other factors unrelated to mastitis (e.g., animal breed, number and stage of lactation, time between milkings, chemical composition of milk), may influence the EC of milk [81,86,88,89,90].

As milk EC is closely related to the physiological and health status of the individual animal, it is not sufficient to use absolute thresholds to detect changes. Instead, conductivity values should be compared with historical data from the same animal and differences analysed between quarters or over several days to identify pathological changes in the udder. Although changes in milk EC can be a useful indicator, they are not always reliable or sensitive enough to make a conclusive diagnosis [91,92].

Therefore, the integration of additional mastitis detection systems, such as somatic cell count (SCC) measurements, a milk colour sensor (the presence of a yellowish colour may indicate an underlying infection, whereas a reddish colour could be due to the presence of blood) or a biosensor to detect specific enzymes (e.g., L-lactate dehydrogenase), may facilitate a more comprehensive understanding.

Lactate dehydrogenase (LDH) has great potential in the detection of clinical mastitis [93] and a biosensor using dry-stick technology with 82% sensitivity is commercially available [94].

One of the latest generations of devices is the NIRS analyser, which can determine milk quality (in terms of fat, protein and lactose concentrations) in real time [95]. The availability of data on high-yielding cows allows early detection of specific diseases such as metabolic disorders, milk changes and mastitis, reducing financial losses and improving animal welfare [96].

### 2.2. Farm IoT Sensors

IoT technologies allow a further advantage, which is real-time monitoring that guarantees automatic, fast, and efficient assistance in case of machine failure. Additionally, the implementation of robotic automation solutions has the potential to enhance productivity and foster competitiveness. The development of automation in dairy farming has led to a wide range of monitoring and control applications. These include using sensors for herd management, milk production optimisation, feed distribution efficiency and environmental control [97].

#### 2.2.1. Automatic Milking System (AMS)

Automatic milking systems (AMSs) can optimise the milking process and cow management compared to conventional milking systems [98]. The introduction of AMSs represents a breakthrough in dairy farming, as this system is not simply a replacement for a conventional milking parlour, but rather a new approach to managing a dairy farm [99].

AMSs change milking and the farmer’s schedule, feeding and housing management [100], allowing cows to voluntarily visit the robots several times a day. This increases milk production and improves animal welfare [100,101,102].

AMSs integrate sensor technology and employ M2M communication to automate many aspects of farm management, such as measuring and distributing concentrated feed based on the stage of lactation and milk production levels and monitoring milk quantity and quality at different scales (from the single udder quarter to the entire cow). This sensor technology application can also provide information about physiological parameters linked to animal health (milk somatic cells, milk colour and conductivity, composition of milk, rumination activity) and herd fertility (heat activity).

Thousands of data points are gathered daily by remote sensors fitted into the milking robot arm and into wearable devices (e.g., cow collar and pedometer), sent wirelessly to a network where they are next routed, often through the Internet, or to a server such as a personal computer or, more commonly, stored in the cloud. At this point, the data are analysed and acted upon according to the specific software in place, allowing deviations from the reference performance to be identified and the herdsman to be alerted to cows that require special attention (management by exception). IoT technologies offer the additional advantage of real-time monitoring of the AMS, ensuring automatic, fast and efficient assistance in the event of machine failure. Finally, an application on a mobile device can be used to check that the AMS is working properly and to switch it off in the event of a problem.

Overall, when evaluating the integration of an automatic milking system (AMS), the specific technical and operational characteristics of the farm should be taken into account. The success of AMS implementation depends firstly on the attitudes and expectations of dairy farmers [103,104,105].

Over the past three decades, AMSs have made it possible to fully automate milking activities, overcome difficulties in finding skilled labour, reduce heavy milking workloads and increase the number of milking events without additional labour costs. These elements, combined with improved animal welfare and increased milk yields, have led to more than 50,000 AMSs being in operation worldwide in 2020 and more than 50% of dairy farms in north-western Europe being equipped with automated milking systems by 2025 [106]. These systems can be used in free-stall and pasture-based environments, and since 2008 robotic milking has been extended to dairy buffaloes on a commercial farm in southern Italy [107]. Recently, robotics tailored to rotary parlours have been trialled in Australia and are starting to spread in Europe, too. Automated rotary systems are suitable for large herds (>500 cows), allowing milking with much higher throughput levels than conventional systems. Like AMSs, they employ sensor technology and M2M communication to identify individual cows and monitor milk quality and animal health.

The latest evolution in automated milking introduces batch milking—a dairy farming practice where cows are milked by AMSs in groups at fixed milking times, typically two or three times a day. Robotic batch milking takes advantage of automated milking technology while allowing producers to manage cows and farm labour in their own way.

#### 2.2.2. Automatic Feeding Systems (AFS)

**Figure 4:**
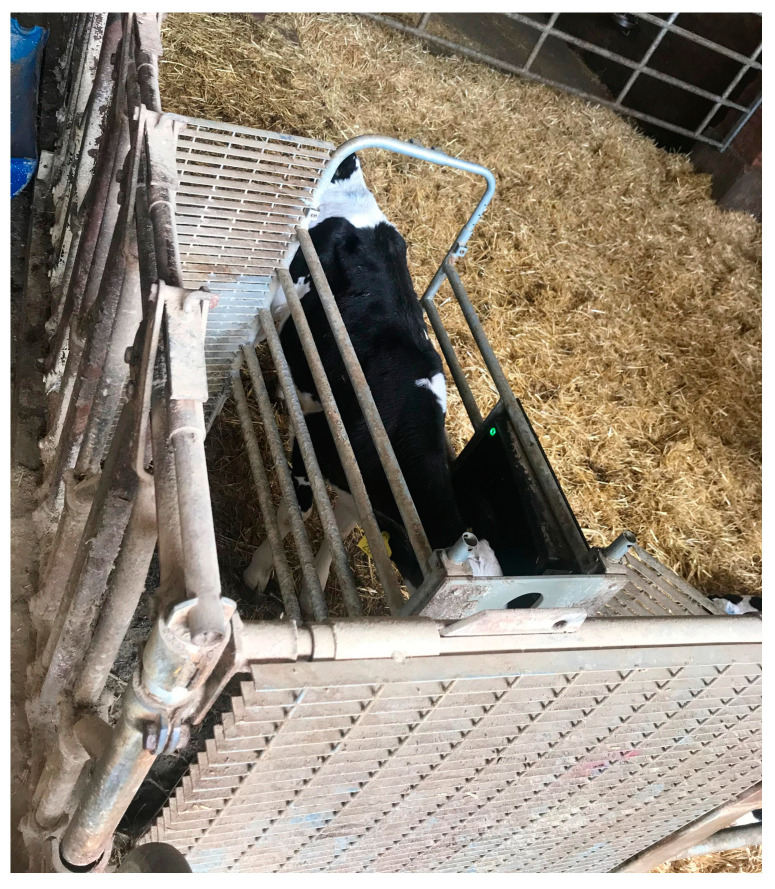
Top-view photo of a calf feeding from an automatic milk delivery system.

Feeding represents a significant financial burden on dairy farms, accounting for up to 50% of total operating costs, and is the second most time-consuming task after milking. Automation of feeding practices has mainly involved the use of automatic concentrate feeders for cows to address nutritional deficiencies that cannot be met by a total mixed ration, while self-feeders for calves ensure that each animal receives a precise ratio of feed tailored to its nutritional requirements (Figure 4). Incorporating these technologies reduces the time needed to prepare and distribute feed, while increasing the ability to monitor the health of individual cows and calves.

In the 2000s, advances were made in automated feeding systems designed for TMR and partially mixed rations (PMR) [108,109]. These systems remove the need for farmers to prepare and deliver TMR or PMR and allow programmable feed distribution and more frequent daily feeding. Recent studies have shown that this technology can reduce reliance on manual labour, increase flexibility of work schedules [110], encourage cow feeding activity, increase dry matter intake and promote natural feeding behaviour by providing meals more regularly [111,112,113,114,115].

Most robotic feeding systems consist of feed bunkers that are typically refilled every 1–3 days. The feed is automatically loaded into a stationary mixer where the TMR is prepared and then distributed to the cows by rail-mounted distribution wagons. Other AF systems are based on self-contained, battery-powered mixing and feeding robots.

The weighing and programming of TMR are controlled by a dairy farm management system that connects the different barn elements (milking, feeding, monitoring, and sorting equipment) into a network, according to the IoT logic. This system offers full control over TMR management, enabling the handling of feed components, recipes, animal groups, and batches. Other benefits include access to feeding history, stock control and assessment of economic evaluations related to feed composition, nutrient content and costs associated with variations in milk production volumes. This ensures better control over feed efficiency, facilitated by specific web and smartphone applications that are usually connected to the cloud where the data are stored.

NIR sensors enable non-disruptive, instant monitoring of ration composition, which has proven to be very useful in mitigating seasonal fluctuations and minimising differences between feed and rations [116]. In Tangorra et al. [117], a NIR diode was employed to calculate the homogeneity of the TMR produced in the tank of a mixer wagon. The application of NIR technology facilitates the consistent production of TMR, accompanied by cost and waste reduction and an enhanced economic efficiency and yield of the system [118].

Optical sensors, consisting of cameras that take multiple frames during mixing, are also employed for characterising the TMR, including measuring fibre length and ration homogeneity. These sensors minimise the potential for operator error by ensuring the provision of a more suitable feed.

Automatic feed-pushing systems can reduce feed sorting. This tool has revolutionised farming activities by eliminating the need for manual re-pushing in the barn. Feed pushing is essential to increase animal intake [119,120].

**Figure 5:**
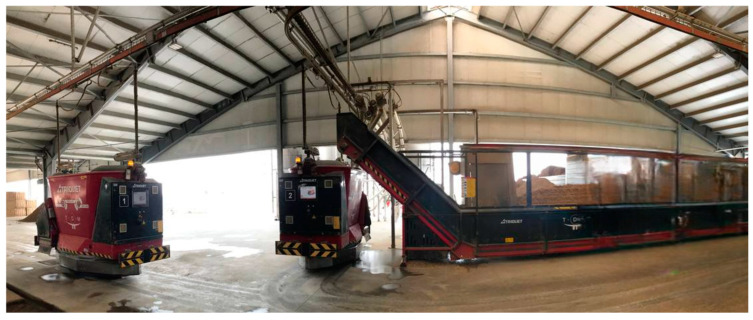
Example of commercially available automatic feeding system solution.

The last automated feeding system realized represents the highest level of automation currently available. These devices can undertake tasks such as ration preparation, distribution, and re-pushing (Figure 5). Over 1250 automatic feeding robots have been installed to date [121].

Each system comprises a kitchen and a distribution robot. The kitchen stores the raw materials required for ration production, and in the most advanced models the cutting and preliminary preparation of raw materials can also be conducted autonomously by automated system. Automatic distributors can be classified into three categories based on the level of automation achieved. The lowest level of automation is limited to distribution and mixing. The second level is capable of self-loading feed. Finally, the third-level AFS exhibits a comprehensive degree of automation, encompassing all necessary activities [119].

Automated feeding systems are transforming the dairy farming industry, boosting efficiency, reducing labour costs and improving animal health and productivity. As technology continues to advance, these systems are likely to become an increasingly integral part of modern dairy operations, providing a sustainable solution to the challenges of feed management.

#### 2.2.3. Environmental Quality Sensors

Environmental sensors on cattle farms play a pivotal role in monitoring climatic and environmental conditions, including temperature, humidity, and air quality. The data obtained from these sensors facilitate maintaining an optimal microclimate for the welfare and health of cattle, reducing the risk of heat stress and other climate-related issues.

Heat stress disrupts animal homeostasis due to excessive temperature, resulting in higher-than-normal body temperature and increased respiratory and heart rate [122]. This multifaceted condition affects various physiological functions, including metabolism, the endocrine system, the immune system, and reproduction [123,124]. According to several studies, heat stress significantly impacts cattle metabolism and immune function, leading to a decline in overall health and productivity [123,124].

In response to heat stress, food intake decreases due to the direct negative impact on the hypothalamus’ appetite centre and the animal’s attempt to minimize endogenous heat production [125,126].

In addition, heat exposure alters the composition of the ruminal microbiome, reducing buffering capacity, which leads to an increase in lactate production. This metabolic imbalance, combined with a reduction in dry matter ingestion, results in an acidification of the rumen pH, which raises susceptibility to metabolic disorders [127].

Heat stress adversely affects rumination time, which is closely related to milk production. During stress, the concentration of fat and protein in milk decreases due to a decrease in protein synthesis and an increase in somatic cells [128,129]; both milking duration and time spent in the milking parlour decrease, further contributing to reduced production [130]. Therefore, it is imperative that appropriate measures are taken to manage and mitigate the damaging effects of these adverse climatic conditions.

Barn design and natural ventilation are fundamental from a structural and plant engineering perspective. Farms should be designed with appropriate orientation to promote natural ventilation and improve structural aeration [131,132].

**Figure 6:**
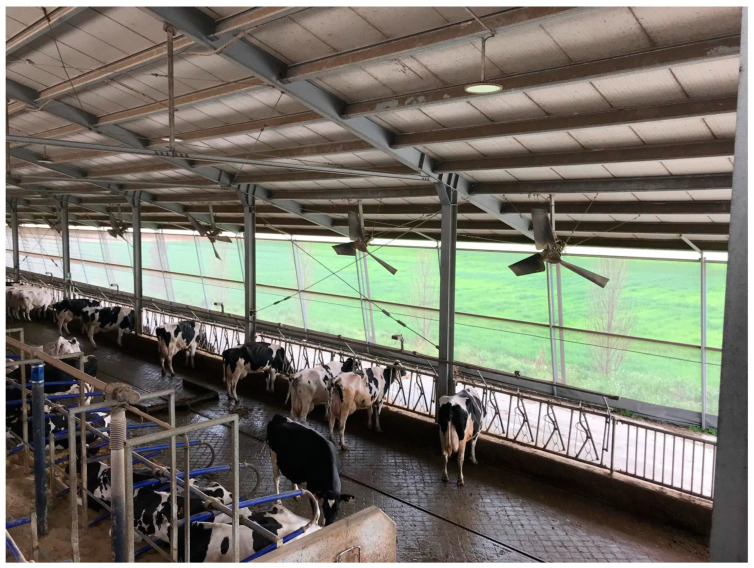
Cooling system applied in an Italian dairy farm equipped with high-speed fans and low-pressure foggers.

An example of a typical cooling system applied in a dairy farm can be observed in Figure 6.

Convective cooling, using high-speed fans designed for low air volume (LVHS) and low-speed fans meant for high air volume (HVLS), can help in reducing barn temperature [133]. The combination of fans and wetting systems has shown to improve ingestion, boost production, and enhance animal comfort [134].

In hot dry climates, high-pressure foggers reduce the ambient temperature by releasing small water droplets, while low-pressure foggers with large water droplets are optimal for climates with high relative humidity [135].

A well-regulated cooling system controlled by the Temperature-Humidity Index (*THI*) effectively allows management of heat stress in dairy cows.

The *THI* index, which considers dry temperatures, wet bulb temperatures, and relative humidity on livestock, is fundamental in assessing heat impact on cows [122,136]. The *THI* is calculated in several ways, but the most recognized formula is as follows (1):

(1) $${THI}_{ijk}=\left(1.8\times {ET}_{ijk}+32\right)-\left(0.55-0.0055\times {RH}_{ijk}\right)\times \left(1.8\times {ET}_{ijk}+32\right)-58$$

where *ET* is the environmental temperature (°C) and *RH* is the relative humidity (%) [137]. The threshold value for animals’ welfare is around 68, but can vary based on factors like breed, age, and lactation number [138].

This system combines wetting and force ventilation to promote heat dissipation by evaporation, which is more efficient and economical than indirect cooling.

**Figure 7:**
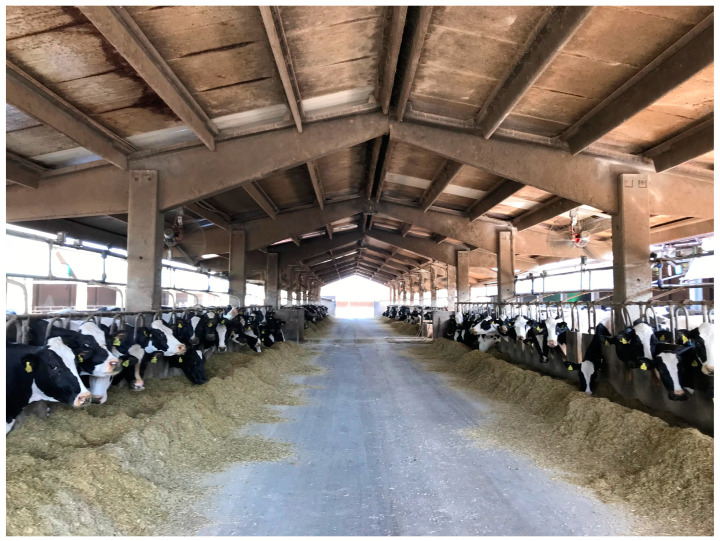
Sprinkler systems installed above the cow feeding area in a typical Italian farm.

Sensors along the feeding aisle detect cow presence during mealtimes and when the *THI* exceeds the threshold value of 68, foggers moisten the animals, followed by fan activation to facilitate evaporation and maintain optimal surface temperature. Wetting, pausing, and fan activation times are automatically adjusted according to *THI* levels, ensuring optimal cooling and animal comfort (Figure 7).

The assessment of environmental conditions in cattle farms is conducted using different types of sensors. While *THI* is determined solely by temperature and relative humidity, a comprehensive and accurate evaluation of ambient environment requires monitoring of additional parameters, including the following:

- Temperature (°C), measured using a thermometer. It is the most critical parameter for monitoring ambient temperature to avoid extreme heat conditions.
- Relative humidity (%), assessed using a hygrometer. When combined with temperature data, provides a clearer understanding of heat stress risks and *THI* Index.
- Air velocity (m/s), measured using an anemometer (3D anemometer can also measure wind direction). It is fundamental for evaluating ventilation efficiency and ensuring proper airflow in barns.
- Solar radiation (W/m^2^), monitored with a pyrheliometer. It helps in assessing the impact of direct sunlight on animals, especially in outdoor or semi-open farm environments.

The use of weather stations enables correlation of indoor and outdoor conditions allowing for more precise management of open or semi-open naturally ventilated buildings. These sensors continuously collect various environmental data and send them to a control unit to adjust cooling units. This type of automated system ensures precise intervention when necessary, reducing water and energy wastage.

The development of sophisticated automated monitoring techniques has enabled the integration of sensors for environmental monitoring with other physiological parameters of animals, providing detailed real-time information on individual animals. Parameters examined include rectal temperature [122], deep body temperature, e.g., vaginal temperature [139], the utilisation of thermo-recorders implanted in the skin, rumen temperature sensors, infrared thermography and milk temperature recording [140], as well as heart rate, respiration rate, sweating rate, and lying patterns of the animals. This integration enhances the ability to predict heat stress and respond with tailored interventions.

Moreover, cameras used for imaging analysis can offer additional insights into animal behavioural patterns, such as feeding and drinking activities, which have been demonstrated to be strongly related to heat stress conditions. By combining environmental and behavioural data, farms can more accurately assess cattle welfare and take preventive measures.

Finally, the integration of environmental sensors with data management systems enables predictive analysis and timely intervention, improving production efficiency and sustainability [135]. By anticipating heat stress events and automating cooling measures, farms can minimize production losses and optimize resource use, contributing to both animal welfare and farm profitability.

## 3. Discussion

Automatic and IoT technologies enable the control and management of larger herds by providing detailed information on each cow. Precision Dairy Farming (PDF) involves using advanced technologies to monitor and manage cows individually, enhancing farm efficiency and animal welfare.

As defined by [141], PDF is “the use of information and communication technologies for improved control of fine-scale animal and physical resource variability to optimise economic, social, and environmental farm performance”. Similarly, Bewley [142] describes PDF as the use of new technologies to measure physiological, behavioural, and production indicators of individual cows to improve management strategies and farm performance.

**Figure 8:**
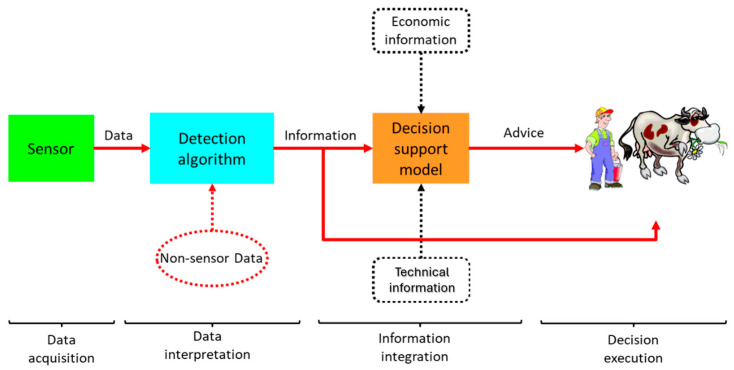
From sensor data to decision-making in a dairy farm management system (modified from [42]).

PDF systems use sophisticated technology to collect data that enables farmers to make data-driven decisions. Figure 8 outlines the different stages of a typical PDF system [42,143]: (i) data acquisition, where sensors monitor and collect specific animal-related parameters; (ii) data interpretation, where specific algorithms process the data collected in the previous stage and extract meaningful insights (e.g., reduced rumination combined with a rise in temperature may indicate illness or imminent calving). These can be enhanced by historical records and non-sensor data [144]; (iii) information integration, where the data processed in stage (ii) is combined with other relevant information (technical, economic, etc.) to support management decisions; (iv) decision execution, where, based on the information, actions are recommended and executed autonomously by the farmer or the system (e.g., the system can adjust feeding strategies or trigger a medical intervention based on health indicators).

Effective data interpretation relies on algorithms that transform raw data into meaningful insights. This process requires a clear definition of the animal or farm status to be monitored and an established standard for comparison. The challenge is that there is considerable variability among individual cows, which makes signal interpretation more difficult.

However, the variability among cows can make signal interpretation complex. For example, current models for detecting illnesses often struggle with false alarms due to diverse data inputs. This trade-off between sensitivity and specificity [42] highlights the challenge of achieving accurate diagnoses.

Making informed decisions depends on the system’s ability to apply suitable management strategies based on the data [145]. False positives or negatives can reduce the effectiveness of interventions, making the system’s precision critical for practical use.

The success of PDF systems depends on their cost-effectiveness and socio-economic impact. While many PDF systems focus on improving disease management (e.g., mastitis), increasing production efficiency (e.g., automatic feeders), and reducing labour (e.g., automated milking), these improvements must be balanced against the financial investment required [145,146]. For farmers, the long-term benefits—such as enhanced herd health, improved production, and labour savings—must justify the initial setup and ongoing operational costs.

The PDF approach incorporates a comprehensive range of technologies designed to optimise the efficiency, productivity and welfare of dairy cattle [147]. Integrating data from milking, feeding, and monitoring systems offers greater potential. Using a computerized information system to combine and interpret data from sensors, databases, and models enables farmers to maximize the value of the information collected [40,148]. For example, integrating milking and feeding systems with health monitoring allows farms to detect critical issues or optimize feeding strategies across the herd. Comprehensive farm management tools like Lely Horizon (https://www.lely.com/gb/solutions/farm-management/horizon/, accessed on 14 October 2024) or DeLaval DelPro Farm Management (https://www.delaval.com/en-gb/, accessed on 14 October 2024) streamline decision-making, facilitating improvements in production quality, cost reduction, and feed optimization. Additionally, multi-criteria analysis (MCA) tools can help refine farm strategies, balancing different factors to enhance overall farm performance.

From this perspective, PDF should go beyond monitoring technologies to include automated and mechanised systems that improve dairy management processes. These technologies are increasingly moving towards online platforms, using reliable network solutions to provide better services to users [149]. This trend marks the growing role of the “Internet of Things” (IoT) in modern dairy farming, with increasingly connected systems enhancing farm management capabilities.

However, in the agricultural context, several limitations hinder the diffusion of IoT systems, including digital infrastructure and connectivity, the costs associated with adopting new technologies, and the skill levels of farmers [26]. Worker training is needed to develop the capacity to manage and analyse the data collected through IoT technologies. This new approach significantly facilitates herd monitoring and management practices [150]. The Smart Farming approach maintains high animal welfare standards to increase animal production performance and longevity. This is achieved through data collection technologies that, when properly processed, enable the early detection and prevention of potential issues [151].

Further advancements are needed to prioritize data protection and cybersecurity, emphasizing the importance of implementing robust security measures to safeguard valuable information [26]. As technology advances in the agricultural sector, it is important to ensure data protection to maintain consumer confidence and uphold the integrity of dairy operations. Proactive measures must be taken to address these challenges and effectively mitigate potential cybersecurity threats.

## 4. Conclusions

This paper presents an overview of new technologies potentially relevant to dairy farms, including automated milking systems (AMSs), cow monitoring sensors, automated feeding systems and environmental quality sensors. The integration of these technologies within the Internet of Things (IoT) represents a transformative shift in dairy farming, enabling farmers to increase herd productivity, improve animal health and control production cycles proactively, compared to the traditional reactive approaches prevalent in conventional dairy farming.

Precision Dairy Farming (PDF) revolutionises dairy management by enabling individual cow monitoring, optimising feeding and health strategies and reducing labour requirements. The success of these systems depends not only on the quality of the data collected but also on the seamless integration of different systems and their cost-effectiveness. By strategically aligning technology investments with practical farm needs, PDF offers farmers the potential for improved efficiency, productivity and sustainability. Studies have shown that farms using these smart technologies can achieve productivity gains, underlining their importance.

However, several constraints hinder the widespread adoption of IoT systems in agriculture, so it is crucial to prioritise the digitisation of farms and implement robust security measures to protect sensitive data to ensure consumer trust and the long-term success of these innovations in the dairy industry.

## Data Availability

Data are contained within the article.
